# Comparative analyses of longissimus muscle miRNAomes reveal microRNAs associated with differential regulation of muscle fiber development between Tongcheng and Yorkshire pigs

**DOI:** 10.1371/journal.pone.0200445

**Published:** 2018-07-11

**Authors:** Yu Xi, Huijing Liu, Yuqiang Zhao, Ji Li, Wenchao Li, Guorong Liu, Jiayong Lin, Wanghong Liu, Jinlong Zhang, Minggang Lei, Debin Ni

**Affiliations:** 1 Key Laboratory of Agricultural Animal Genetics, Breeding, and Reproduction, Ministry of Education and Key Laboratory of Swine Genetics and Breeding, Ministry of Agriculture, Huazhong Agricultural University, Wuhan, P.R. China; 2 Swine Breeding Quality Supervision and Inspection Center of the Ministry of Agriculture (Wuhan), Huazhong Agricultural University, Wuhan, P.R. China; 3 National Engineering Research Center For Livestock, Huazhong Agricultural University, Wuhan, P.R. China; University of Massachusetts Medical School, UNITED STATES

## Abstract

Tongcheng (TC) and Yorkshire (YK) are two pig breeds with distinctive muscle morphology. Porcine microRNAome (miRNAome) of the longissimus muscle during five developmental stages (40, 55, 63, 70, and 90 days post coitum (dpc)) was explored by Solexa sequencing in the present study to find miRNAs involved in the different regulation of skeletal muscle development between the two breeds. A total of 320 known porcine miRNAs, 64 miRNAs corresponding to other mammals, and 224 potentially novel miRNAs were identified. Principal component analysis (PCA) and hierarchical cluster analysis (HCA) suggested that the factor “pig breed” affected the miRNA expression profiles to a lesser extent than the factor “developmental stage”. Fifty-seven miRNAs were differentially expressed (DE) between the neighbor developmental stages in TC and 45 such miRNAs were found in YK, 34 in common; there were more down-regulated stage-DE miRNAs than up-regulated. And a total of 23, 30, 12, 6, and 30 breed-DE miRNAs between TC and YK were identified at 40, 55, 63, 70, and 90 dpc, respectively, which were mainly involved in cellular protein modification process, protein transport, and metabolic process. As the only highly expressed breed-DE miRNA found in no less than four developmental stages, and also a stage-DE miRNA found both in TC and YK, miR-499-5p could bind the 3’-UTR of a myofibrillogenesis regulator, destrin/actin depolymerizing factor (DSTN), as validated in dual luciferase reporter assay. The results suggested that miR-499-5p possibly play a noteworthy role in the breed-distinctive porcine muscle fiber development associated with the regulation of DSTN.

## Introduction

Muscle development in various types of pig breeds is associated with specific differences in the quality of the meat, such as the taste. Tongcheng (TC), a typical Chinese indigenous pig breed, is characterized by slow-growing muscles that result in tender and delicious meat [1]. Yorkshire (YK), an exotic purebred pig breed, has fast-growing muscles that lead to high lean meat percentage and relatively lower meat quality [2]. Analyses of the development differences in skeletal muscles between them may help the identification of the cellular mechanisms underlying meat quality. These analyses may be beneficial for the improvement of pork production.

Muscle growth is influenced by the type, number, and size of muscle fibers. Muscle fibers are divided broadly into two main types according to their myosin heavy chain (MyHC) composition as well as morphological, biochemical, and physiological properties: type I or slow-twitch oxidative (SO) fibers (for the maintenance of posture and/or endurance exercise) and type II or fast-twitch fibers (for rapid bursts of activity) [3]. The fast-twitch fibers are subdivided into three classes: fast-twitch oxidative (FO), fast-twitch glycolytic (FG), and fast-twitch oxidative glycolytic (FOG), or namely types IIa, IIb, and IIx, respectively, based on their metabolic activities [4–7]. Previous studies have shown that meat quality is positively correlated with the percentage of red skeletal muscles (types I and IIa), while it is negatively correlated with the percentage of white skeletal muscles (types IIx and IIb) [7]. Therefore molecular mechanisms regulating the formation and transition of muscle fibers with time may greatly contribute to meat quality and muscle growth rate.

With regard to the time course of skeletal muscle development in pigs, there are two waves of fiber generation emerging at distinct embryonic stages [8]. The primary myofiber formation starts at around 35 days post coitum (dpc), proceeds until 60 dpc, and disappears at 90 dpc [3, 9]. The formation of the secondary muscle fibers around the surface of the primary myofibers occurs at approximately 54–60 dpc and proceeds until 90 dpc [10]. Some transitions exist between SO and FG fibers and occur from birth to 60 days of age [11–13]. Following 90 dpc, the development of muscle mainly depends on the growth in size rather than the increase in number of fibers [9]. Several genes are known to be key genes in muscle growth, such as MRFs [14], MEF2 [15], Pax family [16–18], and Myostatin [19].

Accumulating evidence suggests that more than 50 microRNAs (miRNAs) are involved in the muscle development [20–22]. miRNAs are non-coding RNA molecules and play important roles in many biological processes [23]. In animals, the lengths of miRNAs are estimated to 22 nucleotides (nt) and miRNAs exerts direct translational repression rather than mRNA cleavage [24]. It was also reported that target mRNA degradation provides a major contribution to silencing by miRNAs [25]. Nevertheless, it is still unknown which is the main way of miRNA regulation of gene expression in animals, but both two ways may coexist. MiR-1, miR-133, and miR-206 are muscle-specific miRNAs [26–29]. Several research groups have studied miRNAs by sequencing methodologies in order to identify the potential functional miRNAs during skeletal muscle development in pigs [20, 21, 30–33], but a limited number of studies have compared the miRNAs involved in the muscle development between different swine breeds during embryonic development.

We previously reported the mRNA profiles in the longissimus muscle during 11 developmental time points for TC and YK pigs (30, 40, 55, 63, 70, 90, and 105 dpc, and 0, 7, 21, and 35 days postnatal) [29]. In the present study, in order to further explore the molecular network underlying muscle growth from the perspective of post-transcriptional regulation, we evaluated the difference of miRNAs expression between TC and YK breeds at these five key stages [34] during the porcine skeletal muscle development, using the Solexa sequencing technology. This work aimed to provide additional explanations regarding differences of meat quality between TC and YK breeds, and improve our understanding of the miRNA involvement in muscle development.

## Materials and methods

The protocol was deposited in protocols.io (dx.doi.org/10.17504/protocols.io.mpkc5kw).

### Ethics statement

All experiments involving animals were conducted according to the relevant guidelines and regulations and approved by the Ethics committee of Huazhong Agricultural University, Wuhan City, Hubei Province. All the experimental animal procedures were performed according to the Recommendations from the Hubei regulations for the Administration of Affairs Concerning Experimental Animals, 2005.

### Animal challenge, small RNA library construction, and sequencing

Fifteen purebred TC sows and fifteen purebred YK sows [8] with similar age, weight, and genetic background were obtained from the pig breeding farm of Huazhong Agricultural University (Wuhan, China). They were cared for and housed in the same conditions, according to the relevant guidelines and regulations mentioned in the Ethics statement. Pigs had free access to food and water. The gilts were artificially inseminated with the breed-respective semen from the same purebred boars. After a warm shower to relax the pigs, they were stunned with a low-voltage electric shock to reduce the pain from slaughter. The pregnant sows were slaughtered at 40, 55, 63, 70, and 90 dpc for each breed. The fetuses were extracted and the longissimus muscles were dissected from the fetuses. The female fetuses were selected at 55, 63, 70, and 90 dpc, and sex was ignored at 40 dpc, for it had been reported that sex scarcely showed an independent impact on muscle fiber development at the early stage [35]. Thirty muscle samples were obtained and prepared. All samples were immediately snap-frozen in liquid nitrogen and stored at -80°C until use. The total RNA from each sample was extracted using Trizol (Invitrogen, Carlsbad, CA, USA) following the manufacture’s recommendations. RNA quality and quantity were evaluated using the Agilent 2100 Bioanalyzer. All RNA samples exhibited 28S/18S >1.5 and RNA integrity numbers (RIN) >8.0. Equal quantities of isolated RNA were pooled from three individual fetuses from different sows at each time point. We constructed 10 small RNA libraries based on the standard Illumina protocols. The libraries were sequenced on an Illumina Genome Analyzer (Beijing Genomics Institute (BGI)). The 50-nt sequence tags analysis based on Illumina HiSeq TM 2000 high-throughput sequencing uses the SBS-sequencing by synthesis.

### Alignment and annotation of small RNA

The raw data were filtered using the following processes: A) discarding low-quality reads; B) trimming the adaptor sequences; and C) eliminating sequences smaller than 18bp and reads with no insertion. Then, the clean reads were obtained and mapped to the genome by SOAP [36] to analyze their expression and distribution on the genome. The reference database of the pig genome was used for mapping (Sscrofa10.2, ftp://ftp.ensembl.org/pub/release-76/fasta/sus_scrofa/dna/Sus_scrofa.Sscrofa10.2.dna_rm.toplevel.fa). A search against miRbase (version 20.00; www.mirbase.org) was conducted for all of the clean reads by BLAST [37]. The reads that could not be mapped were subsequently annotated and classified according to Genbank (www.ncbi.nlm.nih.gov) and Rfam (version 11.0; http://rfam.sanger.ac.uk) databases by BLAST. To make every unique small RNA mapped to only one annotation, we followed the following priority rule: rRNAetc (in which Genbank>Rfam) > known miRNA> repeat > exon > intron [38]. The remaining non-annotated siRNA sequences were analyzed by MIREAP (http://sourceforge.net/projects/mireap/) to predict novel miRNA candidates. The sequences that were located in the porcine genome and could be folded into typical hairpin structures with nearby sequences were considered to be potential novel miRNAs. The novel miRNAs were aligned to mature miRNAs from other mammals in the miRbase by BLAST in order to obtain more information.

### Expression profiles analysis

The miRNA expression profiles were normalized by transcript per million (TPM), as previously reported [39]. Normalization formula: TPM = actual miRNA count/total count of clean reads*1,000,000. If the normalized expression of a certain miRNA was zero, we revised its expression value to 0.01. The miRNAs with expression values of >28 TPM in at least one of the 10 libraries were selected for all the following analyses.

### Cluster analysis

For principal component analysis (PCA) and hierarchical cluster analysis (HCA), the expression values of the miRNAs were normalized using the Z-score prior to their use for PCA and HCA. The analyses were conducted using the R packages of g models.

### Time-series analysis

Short Time-series Expression Miner v 1.3.8 (STEM, http://www.cs.cmu.edu/~jernst/stem/) was used in order to cluster and visualize possible profiles of miRNA expression over time. The maximum number of model profiles was adjusted to 20 and the maximum unit change in model profiles between time points was set to 1. The STEM clustering method was selected and other options were set as default. The miRNA expression profiles were clustered based on statistically significant values (P-value<0.05).

### Differential expression of miRNAs

The analysis of differentially expressed (DE) miRNAs was performed. The absolute value of fold change and P-value were calculated. Fold-changes of miRNA expression between two samples were calculated with log 2 TPM (log 2 ratio). The P-value formula was:

$$\text{p}\left(\text{x|y}\right)={\left(\frac{N_{2}}{N_{1}}\right)}^{y}{\frac{\left(x+y\right)!}{x!y!{\left(1+\frac{N_{2}}{N_{1}}\right)}^{\left(x+y+1\right)}}}_{D\left(y\geq y_{max}\text{|}x\right)=\sum_{y\geq y_{max}}^{\infty }p(y|x)}^{\text{C}\left(\text{y}\leq y_{min}\text{|x}\right)=\sum_{y=0}^{y\leq y_{min}}p(y|x)}$$

where x and y represent TPM of a given miRNA from the same time point between TC and YK, respectively; N1 and N2 represent the total count of clean reads at the time point between TC and YK, respectively [40].

### Target prediction, GO, and KEGG ontology analyses

The targets of miRNAs were predicted using three available target prediction programs: TargetScan (http://www.targetscan.org/), Mireap (http://sourceforge.net/projects/mireap), and miRanda (http://www.microrna.org/). The target genes identified by all three programs were considered to be the predicted target genes for each miRNA. Functional annotation of the predicted miRNA targets was performed based on the Gene Ontology slim database (GO-Slim) and Kyoto Encyclopedia of Genes and Genomes database (KEGG) using PANTHER (http://www.pantherdb.org/) and KOBAS (http://kobas.cbi.pku.edu.cn/). Default settings were used for the statistical overrepresentation test of GO-Slim [34]. The binomial test was used as the statistical method of KEGG Orthology enrichment. The enriched functional categories with P-values <0.05 were defined as significantly enriched in the target gene candidates.

### Interaction network of miRNA-mRNA

The data of gene/mRNA expression patterns during porcine skeletal muscle development in TC and YK were acquired from our previous work [34]. Firstly, breed-specific DE miRNAs found at any of the five developmental stages were respectively paired with their software-predicted gene/mRNA targets (Figure A in S1 Fig), based on the negative correlation (Spearman’s correlation coefficient <0.5) between the expression trends of the miRNA and the gene/mRNA during muscle development in TC and/or YK. Secondly, breed-DE mRNAs (also between TC and YK) found at any of the five developmental stages were selected (Figure B in S1 Fig). Thirdly, the intersection of the set “breed-DE miRNA & target pairs” and the set “breed-DE mRNAs” were considered as the breed-DE miRNA & breed-DE mRNA pairs (Figure C in S1 Fig). Lastly, the interaction network was constructed using these breed-DE miRNA & breed-DE mRNA pairs and was visualized, using the Cytoscape software (http://www.cytoscape.org/). The parameter of degree in Cytoscape was used to define the hub miRNAs and mRNAs.

### Dual luciferase reporter assay

The sequence containing the miRNA binding from 3’UTR of destrin/actin depolymerizing factor (DSTN) was inserted into the pmirGLO Dual-Luciferase miRNA Target Expression Vector (Promega, Madison, WI, USA). The putative miRNA targeting site was varied by 4nt using overlap-extension PCR. The complete plasmids were detected by double enzyme digestion and sequencing analysis. A total of 50 nM of miRNA mimics (GenePharama, Shanghai, China) or negative controls(NC) was co-transfected into PK-15 cells in 24-well plates with wild type or mutated 3’UTR Dual Luciferase plasmid (200 ng) using X-treme GENE HP DNA Transfection Reagent (Roche Molecular Systems, Pleasanton, CA, USA). The cells were harvested 24 h after transfection and Dual Luciferase Reporter Assay System (Promega, Madison, WI, USA) was used for the luciferase activity assay. The relative luciferase activity (firefly luciferase activity/renilla luciferase activity) showed the degree of miRNA binding the 3’UTR of DSTN [41].

### Availability of data and materials

All biological sequence datasets supporting the conclusions of this article are available in the NCBI SRA repository (accession number is SRP062447; http://www.ncbi.nlm.nih.gov/sra/?term=SRP062447).

## Results

### Overview of sequencing data

Ten small RNA expression profiles of the longissimus muscle were generated for the TC and YK breeds at 40, 55, 63, 70, and 90 dpc. A total of 75,328,908 and 66,408,399 clean reads were obtained for the TC and YK breeds, respectively, using high-throughput Solexa sequencing (S1 Table). An estimated 86% to 95% of the clean reads exhibited a length of 21–23 nt and the most abundant size was 22 nt, indicating the typical feature of miRNAs of Dicer-processing in animals (S2 Fig). The majority of the small RNAs were located in chromosomes 3, 6, 7, 9, 13, 17, and X in both breeds (S3 Fig). The size and distribution suggested high quality of the sequencing data.

**Fig 1:**
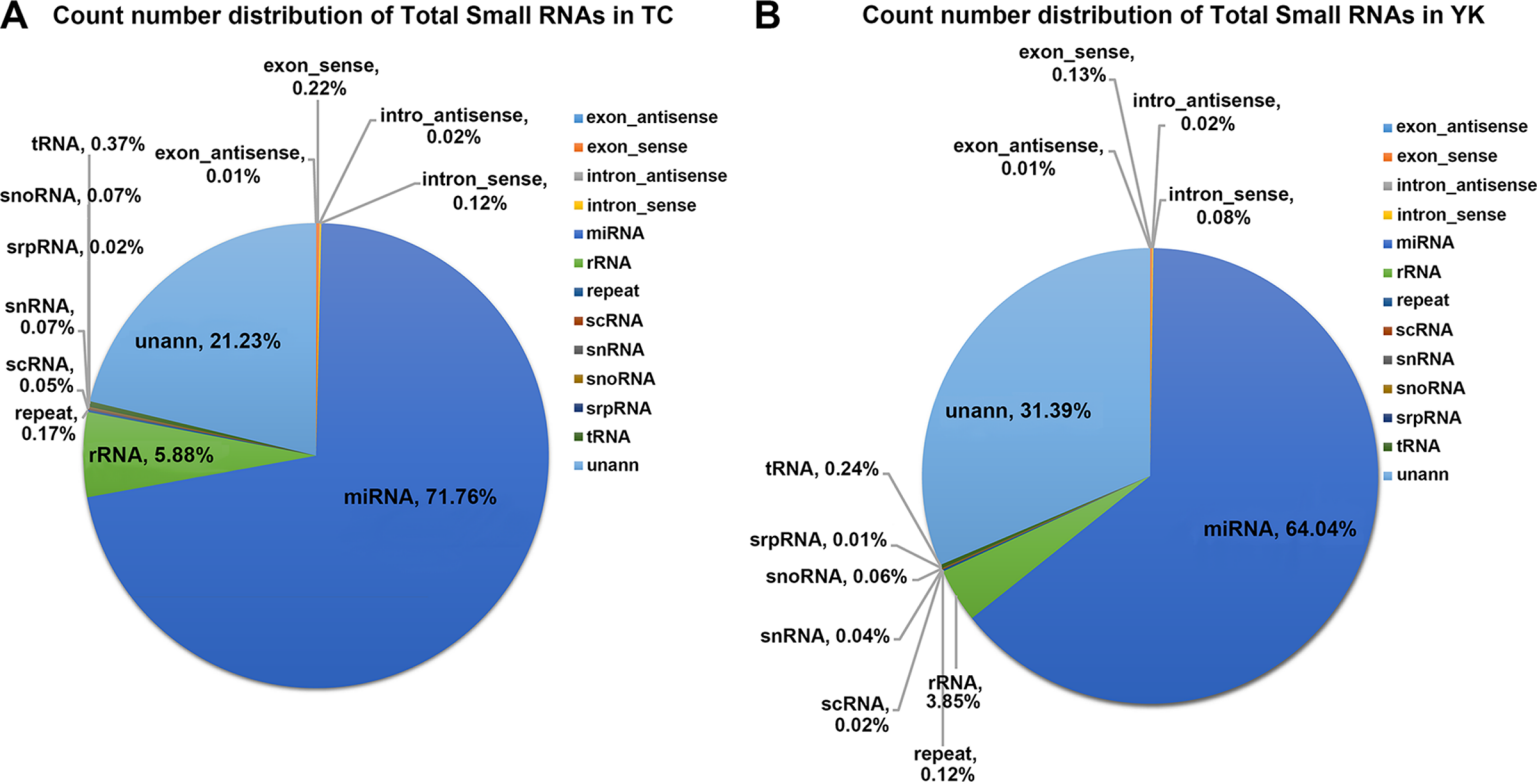
Small RNA annotation. Count number distribution of total small RNA in TC (A) and YK (B) breeds. TC: Tongcheng, YK: Yorkshire. unann: remaining non-annotated small RNAs after searching against miRbase, Genbank and Rfam.

A total of 78.59% and 68.29% reads for the TC and YK breeds, respectively, were mapped to the pig genome (S1 Table) by miRbase-release20, Genbank, and Rfam-release 11.0 database search. The results of the annotation indicated that the porcine miRNAs accounted for 71.76% and 64.04% of the total clean reads in the TC and YK breeds, respectively (S1 Table and Fig 1).

### Annotation and target prediction of porcine miRNAs

The total miRNAs were divided into three categories: 320 known porcine miRNAs, 64 miRNAs corresponding to other known mammalian miRNAs, and 224 potential novel miRNAs (S2 Table). The relative expression levels of the miRNAs were normalized as TPM. The target genes of all miRNAs were predicted from the intersection of Mireap, miRanda, and Targetscan (S3 Table).

### Cluster analysis

**Fig 2:**
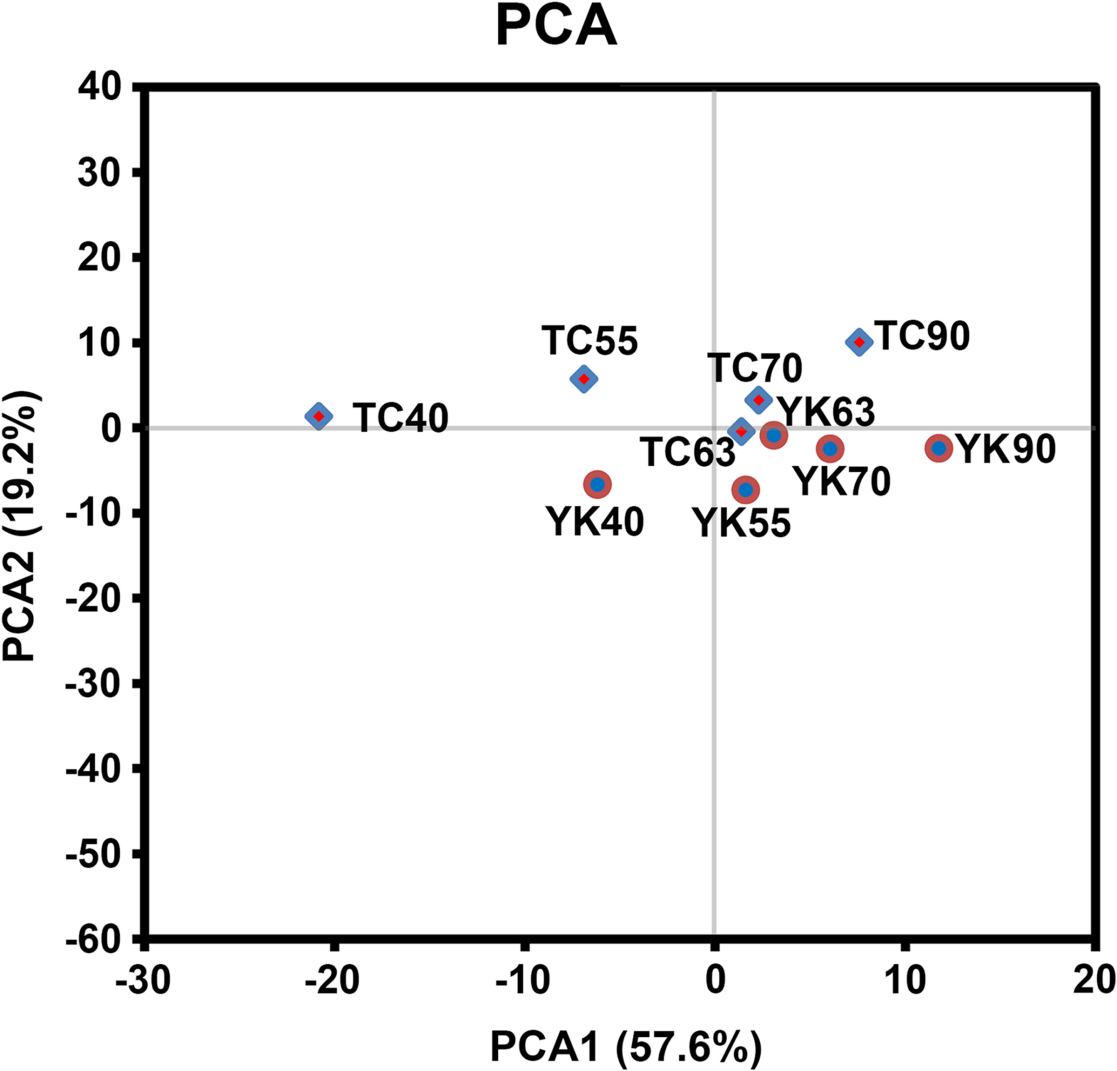
Principal component analysis (PCA) plot of sequencing data in the ten libraries. Clustering of the ten libraries. The rhombuses represent the Tongcheng and the circles represent the Yorkshire breed. TC: Tongcheng, YK: Yorkshire. Stages: 40, 55, 63, 70, and 90 days post coitum (dpc).

**Fig 3:**
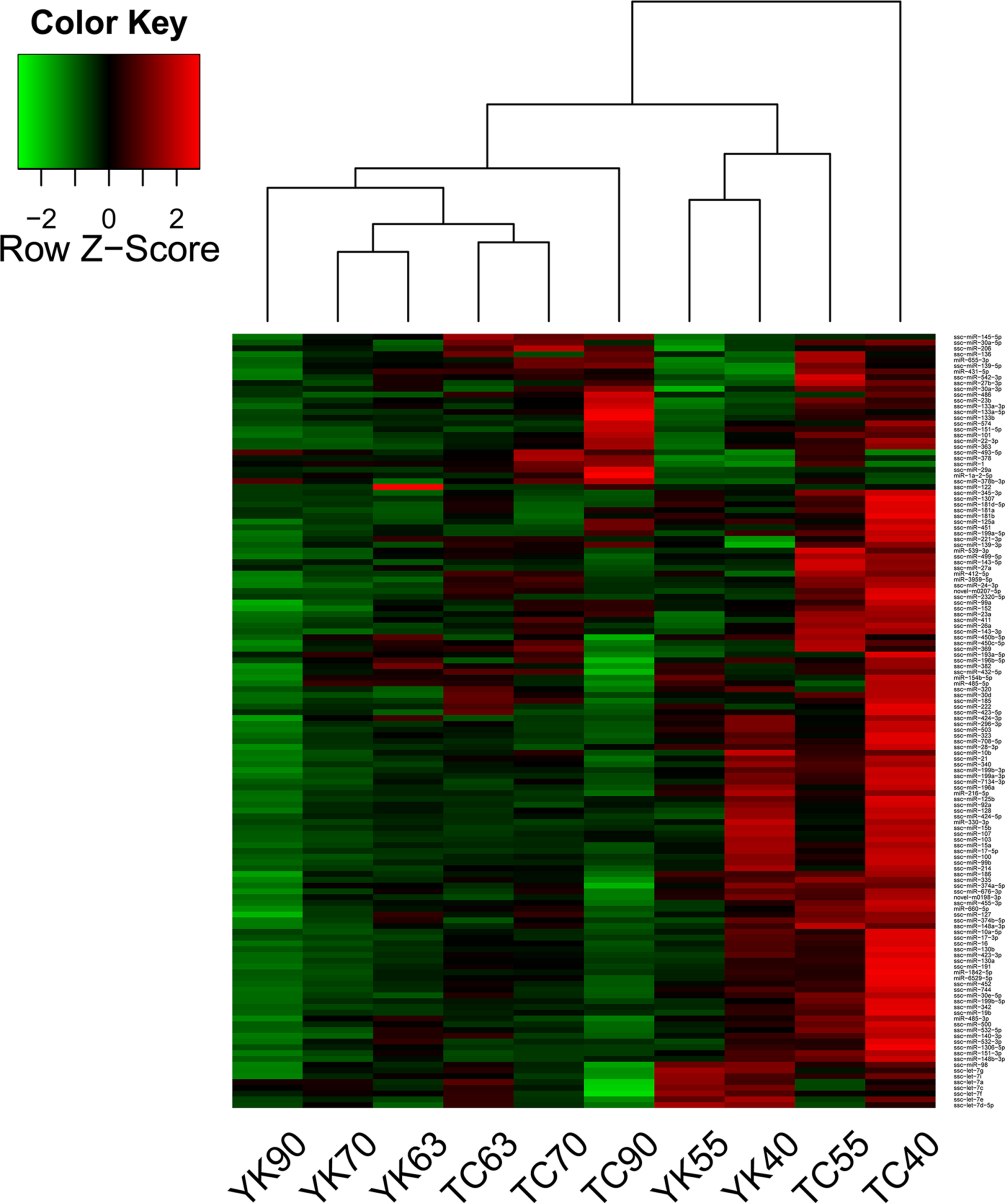
Hierarchical clustering based on the expression of miRNAs. Clustering of the samples was determined by the expression of miRNAs. Red indicates high expression, while green indicates low expression. The expression values were normalized TPM of miRNA using the Z-score. TC: Tongcheng, YK: Yorkshire. Stages: 40, 55, 63, 70, and 90 days post coitum (dpc).

PCA and HCA were used to cluster the samples based on the expression values of 133 miRNAs (S4 Table) [42]. Both analyses produced similar results, indicating that the 10 libraries could be divided into several distinct classes (Figs 2 and 3). The TC40 library exhibited the greater expression dissimilarity from the other libraries, whereas TC63, TC70, YK63 and YK70 were closely clustered together. In general, the clustering results implied that factor “developmental stage” might have a greater impact than the factor “breed” in changing the miRNA expression patterns.

### Time-series expression analysis in TC and YK

**Fig 4:**
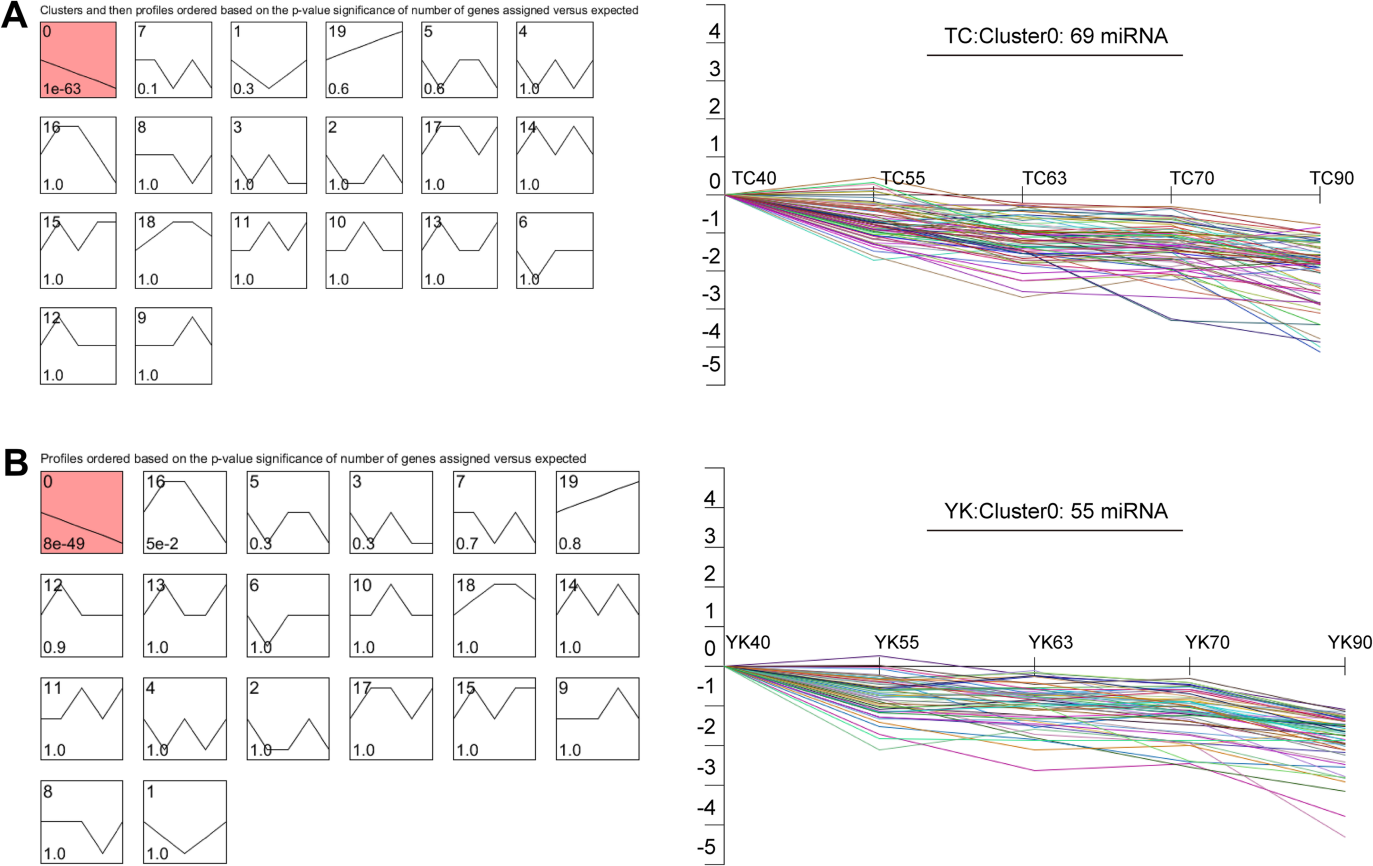
STEM clustering based on differentially expressed miRNAs. Each box corresponds to a kind of expression profile and only colored profiles reach statistical significance. The upper-left number in the box gives information about the order of profile. The bottom-left number gives information about the statistical significance. The right panel displays the patterns of miRNA expression for the “Cluster 0” profile in detail. A: Short time-series expression profiles of miRNAs in TC; B: Short time-series expression profiles of miRNAs in YK. TC: Tongcheng, YK: Yorkshire.

The short time-series expression miner (STEM) was conducted regarding the miRNAs (S4 Table), in order to further explore how “developmental stage” affects the expression patterns of the muscle miRNAs in both the TC and YK breeds. The most typical expression pattern in both breeds was decline over time (Fig 4). A total of 69 miRNAs in the TC breed and 55 miRNAs in the YK breed belonged to the Cluster 0. Interestingly, 47 miRNAs among the55miRNAs of the YK breed were included in the 69 miRNAs of TC. The 69 continually down-regulated miRNAs in TC were mainly involved in metabolic process, protein transport and Chagas disease, while the 55 continually down-regulated miRNAs in YK were mainly involved in metabolic process and protein transport signaling pathway, as indicated by the GO and KEGG analyses (S5 Table).

### Identification of differentially expressed miRNAs among different developmental stages

In addition to the STEM analysis, the screening of stage-DE miRNAs can provide information more specific on how the transition of developmental stages would influence the miRNA expression patterns. Among the highly expressed miRNAs (S4 Table), the DE miRNAs between the neighbor developmental stages (i.e. 40 vs. 55 dpc, 55 vs. 63 dpc, 63 vs. 70 dpc, and/or70 vs. 90 dpc stage-DE miRNAs) were screened in the TC and YK breeds, respectively (S6 Table). A total of 57 stage-DE miRNAs were found in TC and 45 stage-DE miRNAs in YK; 34 stage-DE miRNAs were shared between them (S7 Table and Figure A in S4 Fig). There were more down-regulated stage-DE miRNAs than up-regulated (Figure B and C in S4 Fig).

Functional annotation was performed to the stage-DE miRNAs only in TC or YK, based on their predicted targets (S7 Table). Nineteen GO-Slim biological processes were overrepresented in TC and nine were overrepresented in YK. Interestingly, all the nine GO-Slim biological processes in YK were included in the nineteen GO-Slim biological processes of TC. The 10 GO-Slim biological processes found only in TC were related to metabolic, protein localization, and organization. Besides, 78 KEGG pathways were enriched in TC and 33 were enriched in YK. The KEGG pathway enrichment showed that the pathways found only in TC were involved in the Notch signaling pathway, TGF-β signaling pathway, and TNF signaling pathway, and the pathways only found in YK were involved in Wnt signaling pathway, p53 signaling pathway, and metabolic pathways.

### Identification of differentially expressed miRNAs between two breeds

**Fig 5:**
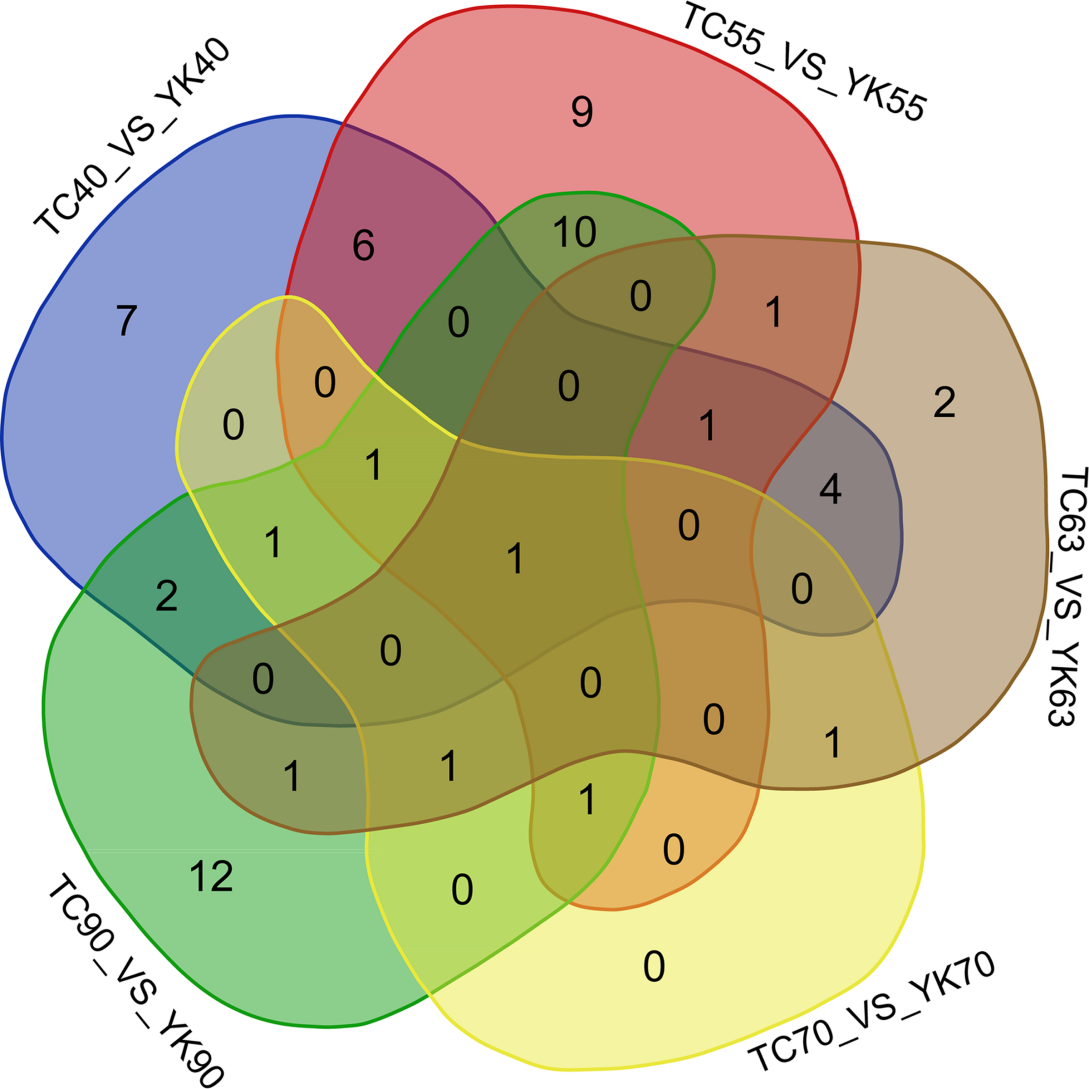
Comparison of differentially expressed (DE) miRNAs among five data sets. The number marked in the overlapping areas shows the common breed-DE miRNAs.TC: Tongcheng, YK: Yorkshire. Stages: 40, 55, 63, 70, and 90 days post coitum (dpc).

The highly expressed miRNAs (S4 Table) were also compared between the TC and YK breeds, at the same time points (TC40 vs. YK40, TC55 vs. YK55, TC63 vs. YK63, TC70 vs. YK70, and/or TC90 vs. YK90). A fold change >2 and P-value <0.05 were defined as the criteria for breed-DE miRNAs (S8 Table). A total of 23, 30, 12, 6 and 30 breed-DE miRNAs were identified in the libraries at 40 dpc, 55 dpc, 63 dpc, 70 dpc and 90 dpc, respectively (S8 Table and Fig 5). Novel-m0207-5p was the only miRNA to be differentially expressed at all five time points (S8 Table), and the varied expressions of ssc-miR-499-5p were detected at four time points (40, 55, 70 and 90 dpc) (S8 Table). Based on the predicted target genes of the miRNAs examined, GO biological process analysis demonstrated that the breed-DE miRNAs were notably involved in cellular protein modification process, protein transport and metabolic process (Table 1), while KEGG analysis indicated that the aforementioned miRNAs were mainly involved in the VEGF signaling pathway, endocrine resistance and phosphatidylinositol signaling system (Table 2).

**Table 1:** GO terms enriched for target genes of breed-DE miRNAs between TC and YK.

RNA metabolic process (GO:0016070)	**	**	**		**
nucleobase-containing compound metabolic process (GO:0006139)	**	**	**		**
nitrogen compound metabolic process (GO:0006807)	**	**	**		**
cellular process (GO:0009987)	**	**	**		**
catabolic process (GO:0009056)		**	**	*	**
cellular protein modification process (GO:0006464)	**	*	*		**
transcription from RNA polymerase II promoter (GO:0006366)	**	*	*		*
protein metabolic process (GO:0019538)	**				**
transcription, DNA-dependent (GO:0006351)	*		*		*
phosphate-containing compound metabolic process (GO:0006796)					**
mRNA processing (GO:0006397)	*				
cellular component organization or biogenesis (GO:0071840)	*				
cell cycle (GO:0007049)		*			
intracellular signal transduction (GO:0035556)			*		

*P<0.05;

**P<0.01

**Table 2:** KEGG orthology enriched for target genes of breed-DE miRNAs between TC and YK.

KEGG_Term	40 dpc	55 dpc	63 dpc	70 dpc	90 dpc
VEGF signaling pathway	*	**	**	*	
Endocrine resistance	*		*	*	*
Phosphatidylinositol signaling system	*	*	*		*
Sphingolipid signaling pathway		**	**		*
Rap1 signaling pathway	*	**	*		
mTOR signaling pathway	*	**			*
Inositol phosphate metabolism		**	*		*
Proteoglycans in cancer		*	**		*
AGE-RAGE signaling pathway in diabetic complications		*	**		*
Longevity regulating pathway—multiple species	*			*	*
Chronic myeloid leukemia	*	*	*		
Platelet activation		*	*	*	
Thyroid hormone signaling pathway		*		*	*
Axon guidance			**	*	
Phagosome	*	*			
Longevity regulating pathway	*				*
Small cell lung cancer	*		*		
Tuberculosis	*	*			
Renal cell carcinoma		*			*
Pancreatic cancer		*			*
T cell receptor signaling pathway		*			*
Phospholipase D signaling pathway		*	*		
Oxytocin signaling pathway			*	*	
Adherens junction			*	*	
Chagas disease (American trypanosomiasis)			*		*
AMPK signaling pathway	**				
Insulin resistance	*				
Endocytosis	*				
Hepatitis B		*			
Leukocyte transendothelial migration			*		
Colorectal cancer			*		
Other types of O-glycan biosynthesis			*		
Glioma			*		
TNF signaling pathway			*		
Fc epsilon RI signaling pathway			*		
Amoebiasis			*		
Notch signaling pathway				*	
Vasopressin-regulated water reabsorption				*	
Spliceosome					*
Synthesis and degradation of ketone bodies					*

*P<0.05,

**P<0.01.

### Interaction network of breed-DE miRNAs and breed-DE mRNAs

**Fig 6:**
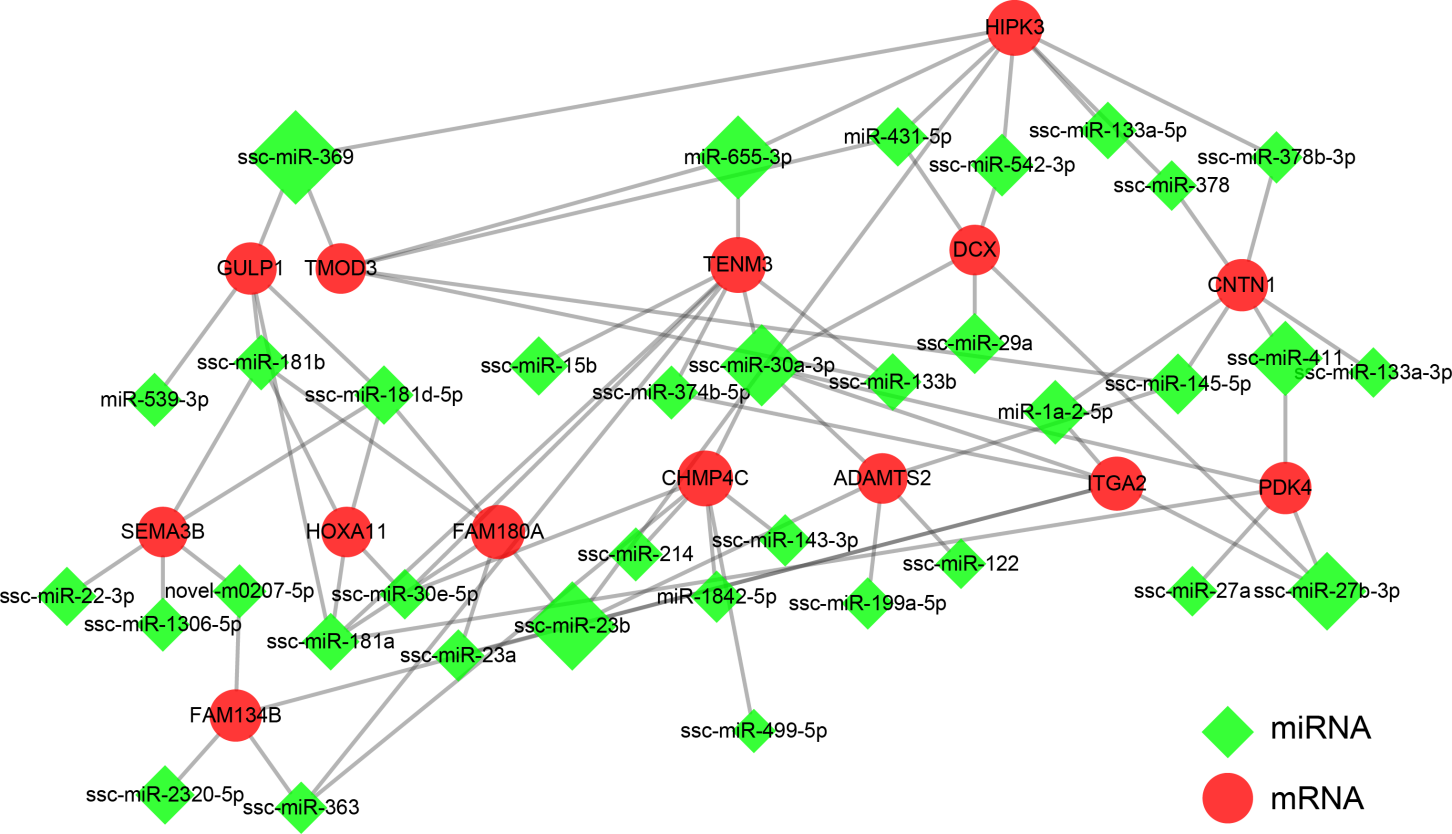
Interaction of breed-DE miRNAs and breed-DE mRNAs. The network was constructed using 36 hub miRNAs and 14 hub mRNAs from the pairs of breed-DE miRNAs and breed-DE mRNAs. The green diamonds represent miRNAs and the red circles represent mRNAs. The nodes with bigger size had higher degree. TC: Tongcheng, YK: Yorkshire. Stages: 40, 55, 63, 70, and 90 days post coitum (dpc).

When combining the temporal expression patterns of miRNAs analyzed in the present work and our previous data of mRNA expression profile in the longissimus muscle tissues of TC and YK pigs during development [34], we obtained 464 pairs of breed-DE miRNAs with breed-DE mRNAs (S1 Fig and S9 Table). In each pair, the breed-DE mRNA was predicted by software as one of the putative targets for the breed-DE miRNA, and it had a negative correlation with that breed-DE miRNAs in the expression trend over developmental stages. The breed-DE miRNA and breed-DE mRNA pairs were used to construct interaction networks (S5 Fig and S10 Table). The whole network was too large for investigation; therefore, we extracted a sub-network (Fig 6), using 35 hub miRNAs and 14 hub mRNAs with degree ≥5 (S10 Table) as the core components. The top three hub miRNAs were miR-369, miR-23b, and miR-655-3p, while the top three hub mRNAs were TENM3, HIPK3, and CHMP4C.MiR-369 was one of the breed-DE miRNAs at 55 dpc; miR-23b, and miR-655-3p were breed-DE miRNAs both at 55 dpc and 90 dpc. TENM3 was differentially expressed between the two breeds at 40 dpc, HIPK3 at 40 dpc and 55 dpc, and CHMP4C at 70 dpc.

### Validation of the target gene of miR-499-5p

**Fig 7:**
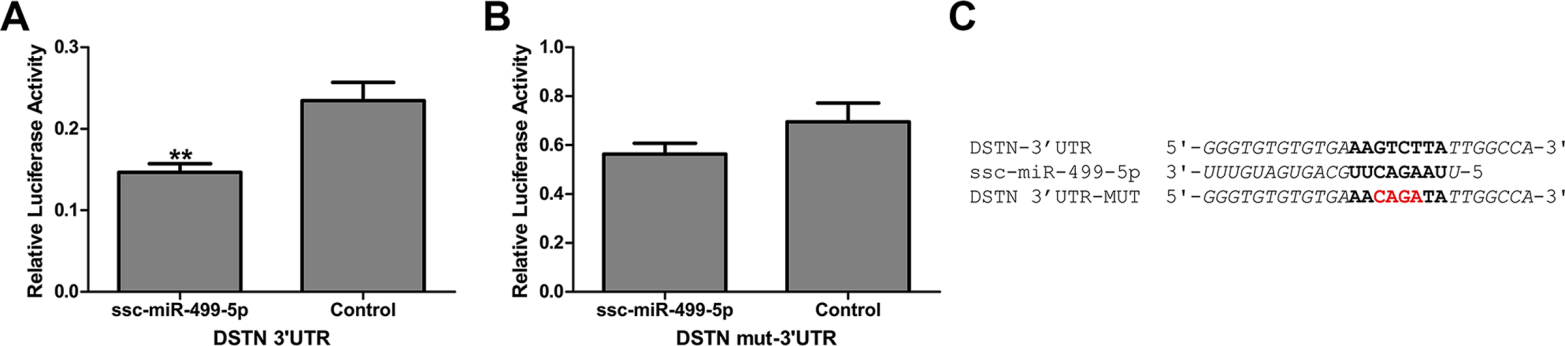
Luciferase reporter assay to validate the miRNA-mRNA relationship of miR-499-5p and DSTN. The analysis of the relative luciferase activity in PK-15 cells following transfection with candidate miRNA mimics. (A)Wild type 3’UTR dual luciferase report vectors were co-transfected with miRNA mimics, which caused a significant decrease in the relative luciferase activity compared with the negative control. (B) Mutated 3’UTR dual luciferase report vectors were co-transfected with miRNA mimics, which did not cause a decrease of the relative luciferase activity compared with the negative control. (C) Predicted target sites of miRNAs and the 4 bp mutation site (red) of genes’ mut-3’UTR. The error bars indicate the SEM. The significance of the differences was calculated using two-tailed T-test. N = 6. **, P<0.01.

It should be noticed that miR-499-5p was the only highly expressed breed-DE miRNA found in no less than 4 developmental stages (S8 Table and Fig 5), and it also was a stage-DE miRNA found both in TC and YK (S6 and S7 Tables). In addition, miR-499 had a degree of 3 in the network of 36 hub miRNAs and 14 hub mRNAs (Fig 6). Taken together, we inferred that miR-499-5p possibly plays a notable role in muscle development regulation, which underlies the meat quality differences between TC and YK breeds. Therefore, we performed preliminary analyses on the regulatory function of this miRNA. The results of the target prediction indicated that the destrin/actin depolymerizing factor (DSTN) was one of the target genes of the miR-499-5p, which was studied in the regulation of muscle development [43]. To validate the predicted relation between miR-499-5p and DSTN, a dual luciferase reporter plasmid containing the miR-499-5p binding sites was conducted. In addition, the luciferase reporters were mutated at the target elements in the 3’UTR of the genes that were the ‘seed’ match region in order to identify the specific binding sites. The primers used for constructing plasmid and overlap-extension PCR are listed in S11 Table. The luciferase activity of the reporter plasmid containing the wild 3’UTRs was significantly suppressed by the miRNA mimics, whereas the activity of the reporter plasmid with the mutated 3’UTRs was not (Fig 7). These results clearly suggested that miR-499-5p directly recognizes and binds to the 3’-UTR of DSTN.

## Discussion

In the present study, the miRNA profiles in the longissimus muscle of the TC and YK breeds at five key stages of fetal skeletal muscle development (40, 55, 63, 70, and 90 dpc) were characterized in order to investigate the roles of miRNAs during muscle development in pigs. A total of 320 known porcine miRNAs, 64 miRNAs corresponding to other known mammalian miRNAs, and 224 potential novel miRNAs were identified. A total of 57 stage-DE miRNAs were found in TC and 45 stage-DE miRNAs in YK; 34 stage-DE miRNAs were shared between them and there were more down-regulated stage-DE miRNAs than up-regulated. A total of 23, 30, 12, 6, and 30 breed-DE miRNAs were identified in the libraries at 40, 55, 63, 70, and 90 dpc, respectively. The breed-DE miRNAs were notably involved in cellular protein modification process, protein transport, and metabolic process. The results also showed that as the only highly expressed breed-DE miRNA found in no less than four developmental stages, and also a stage-DE miRNA found both in TC and YK, miR-499-5p exhibited direct affinity for binding to the 3’-UTR of DSTN, suggesting that miR-499-5p possibly play a noteworthy role in the breed-distinctive porcine muscle fiber development associated with the regulation of DSTN.

Highly abundant miRNAs might play important roles in the regulation of skeletal muscle development or the maintenance of vital physiological functions [31]. Furthermore, a previous study has reported that miRNAs with low expression have no discernible activity [43]. Therefore, in the present study more attention were paid to the highly expressed miRNAs.

The results of PCA and HCA suggested that compared to the pig breed, the developmental stage accounted for a more important proportion of the variance in miRNA expression profile. In other words, developmental stage might play a dominant role in changing the miRNA expression patterns compared with pig breed. Muscle miRNAs of TC and YK had generally similar expression patterns, especially at 63 and 70 dpc. The miRNAs with similar expression patterns in TC and YK might form a fundamental part of the signaling that regulate the muscle development common in pigs. STEM clustering further indicated that the predominant expression pattern of miRNAs in both breeds was sustained decline. A total of 47 miRNAs were down-regulated over time in both breeds. Among these miRNAs, miR-199a-3p showed the highest expression whether in TC or YK (S5 Table). A previous study [44] showed that miR-199a-3p was rich in the skeletal muscles and lung tissues of rat, and its expression ascended firstly and then descended during the myogenic differentiation of C2C12 cell line; when the expression of miR-199a-3p was inhibited in C2C12 cells, the myoblast differentiation was promoted and the size of myotube was increased. The results obtained in the present study and the previous work collectively suggested thatmiR-199a-3p may play a basic role in the early stages of (porcine) muscle development; however, the earlier patterns mediated by miR-199a-3p should be gradually superseded by the following developmental events that impel myogenic differentiation.

Then the influences of the transition of developmental stages on miRNA expression patterns were investigated more in detail by screening stage-DE miRNAs. More stage-DE miRNAs were identified in TC than in YK. The functional annotation also indicated that biological processes in TC were more complicated than in YK, in accordance with the conclusion drawn upon the analysis of mRNA in our previous work [34]. Besides, there were more down-regulated stage-DE miRNAs than up-regulated, consistent with the results of the STEM analysis in the present study and the previous conclusion that a little more up-regulated stage-DE mRNAs were found in mRNA RNA-seq [34].

Furthermore, miRNA expressions were compared between TC and YK in order to get a clear understanding about how the essential development model was characteristically modified in different pig breeds. More breed-DE miRNAs were detected at 40, 55, and 90 dpc. Since there are two waves of muscle fiber formation during the embryonic stages [8], the miRNAs differentially expressed between the two pig breeds at 40 and 55 dpc, but not at 90 dpc, might be involved mainly in the initial muscle development. These miRNAs include miR-2320-5p, miR-431-5p, miR-27a, miR-27b-3p, miR-143-5p, miR-1306-5p and miR-411 (S8 Table and Fig 5). They are not well studies in skeletal muscle development, except for miR-27a. Previous studies showed that miR-27a could decrease myostatin expression, in favor of (porcine) myoblast proliferation [45–48]. And it was discovered that muscle atrophy was associated with low levels of miR-27a [49]. These researches revealed the critical role of miR-27a in driving myogenesis. In the present study, miR-27a expression was higher in TC than in YK at 40 and 55 dpc (S8 Table); and it was one of the hub miRNAs defined in the interaction network of breed-DE miRNAs and breed-DE mRNAs, with a degree of 5 (S10 Table and Fig 6). It could be suggested that the faster formation of muscle fibers in TC as compared to YK [8] might be some extent benefit from the relatively higher expression of miR-27a in the initial stages.

On the other hand, the breed-DE miRNAs observed at 90dpc but not at 40 dpc and 55 dpc might be involved in the mechanisms underlying the growth in muscle fiber size rather than the increase in fiber number [9]. These miRNAs include miR-122, miR-574, miR-29a, miR-151-5p, miR-125a, miR-133a-5p, miR-101, miR-199a-5p, miR-363, miR-532-3p, miR-15b, miR-92a, miR-28-3p and let-7a (S8 Table and Fig 5), ranked in the descending order of the fold change of expression values (TC/YK). MiR-122 and miR-574 were most studied in liver [50] and vascular smooth muscle [51], respectively. While there were some researches focused on the roles of miR-29a in regulating muscle development. It was found that the proliferation of C2C12 myoblast cells was boosted upon miR-29a inhibition [52]; however, the increased expressions of miR-29 and miR-206in C2C12 cells would promote myogenic differentiation possibly by targeting the histone deacetylase 4 (HDAC40) [53], a key suppressor of muscle differentiation. In the present study, miR-29a was found as the only miRNA differentially expressed between TC and YK along from 63 dpc, to 70 dpc, and to 90 dpc (S8 Table and Fig 5); and the fold change (TC/YK) kept rising over time. What’s more, miR-29a was one of the key hub miRNAs in the interaction network of breed-DE miRNAs and breed-DE mRNAs, with a considerable value of 11 in the degree (S10 Table and Fig 6). Therefore, miR-29a presumably plays an important role in mediating the transition from myoblast proliferation to myogenic differentiation, and the gradually higher expression of miR-29a in the muscles of TC than that of YK might contribute to the adequate growth of muscle fibers leading to advantages in meat quality. In addition, A recent study [54] pointed out that a regulator of fat development, C1q/tumor necrosis factor-related protein 6 (CTRP6) could be targeted by miR-29a in porcine adipocytes, suggesting that miR-29a might favor intramuscular adipocyte proliferation through inhibiting CTRP6. Whether the highly expressed miR-29a in skeletal muscles of TC could be secreted extracellularly to affect the intramuscular fat development remains further exploration, which might be another mechanism for the better meat quality of TC.

Finally, the miRNAs differentially expressed between the two pig breeds at all the three time points (40, 55, and 90 dpc) might be deeply involved in the long-term complex regulation that eventually determines the composition of muscle fiber types as well as the meat quality. Such breed-DE miRNAs were novel-m0207-5p and miR-499-5p (S8 Table and Fig 5).The expression level of novel-m0207-5pwas far less than miR-499-5p, and studies about its roles in cell metabolism are lacking. MiR-499-5p was a breed-DE miRNA found at 40, 55, 70, and 90 dpc in the present study, and its expression level gradually down-regulated over time. A recent study showed that miR-499 exhibits a greater expression level in psoas major muscle compared with the longissimus muscle, which respectively have higher proportions of red and white skeletal muscle in pigs [55]. And there was a substantial loss of type I muscle fibers and a concomitant increase in the expression of faster types IIx and IIb muscle fiber in the soleus of double knockout mice for miR-208b^-/-^ and miR-499^-/-^; while the overexpression of miRNA-499 resulted in higher proportions of type I muscle fiber [22]. Taken together, the results suggested that the higher expression of miR-499-5p in TC at several key stages of muscle development probably favor the formation of type I fibers constituting red skeletal muscles, which is positively correlated with good meat quality [7]. The present study also showed that DSTN might be one target of miR-499-5p (Fig 7). DSTN belongs to the ADF family, which is responsible for enhancing the turnover rate of actin and is involved in actin dynamics during myofibrillogenesis [56], thus having an impact on the ratios of different types of fibers. It is plausible that different expression of miR-499-5p in TC might cause changes in the muscle fiber composition as compared to YK, targeting DSTN to fine-tune the actin dynamics. Besides, the predicted hub mRNA target of miR-499-5p was the charged multivesicular body protein 4C (CHMP4C) (S10 Table and Fig 6), which has not been well studied in muscle development until now and deserves some attention in future research.

The present study is not without limitations. The DE miRNA were simply observed and their possible roles were estimated using bioinformatics, but these roles will have to be confirmed in vitro. In addition, many other types of RNAs are involved in the regulation of gene transcriptions. In order to provide a better understanding of muscle development, future studies must use more sequence data and address the combination of the analyses of the protein, mRNA, long non-coding RNAs, circRNA, and miRNAs profiles.

## Conclusions

The present study examined the miRNAs differentially expressed during porcine muscle development between TC and YK. The results are expected to improve the understanding of the distinctive myogenetic regulation mediated by DE miRNAs in TC as compared to YK, and provide implications for the improvement of meat quality in animal production.

## Supporting information

S1 Fig Diagram depicting how to obtain the breed-DE miRNA & breed-DE mRNA pairs. (A) Breed-specific DE miRNAs found at any of the five developmental stages were respectively paired with their software-predicted gene/mRNA targets, based on the negative correlation (Spearman’s correlation coefficient<0.5) between the expression trends of the miRNA and the gene/mRNA during muscle development in TC and/or YK. (B) Breed-DE mRNAs (also between TC and YK) found at any of the five developmental stages were gathered. (C) The intersection of the set “breed-DE miRNA & target pairs” and the set “breed-DE mRNAs” were considered as the breed-DE miRNA & breed-DE mRNA pairs.
(PPTX)

S2 Fig Sequence length distribution of small RNA. TC: Tongcheng, YK: Yorkshire. Stages: 40, 55, 63, 70, and 90 days post coitum (dpc)
(TIF)

S3 Fig Chromosomal location of small RNA. Chromosomal locations of small RNAs are displayed across 20 chromosomes and mitochondrial DNA (MT). TC: Tongcheng, YK: Yorkshire.
(PDF)

S4 Fig The number of differentially expressed miRNAs among different developmental stages. Number of differentially expressed miRNAs. TC: Tongcheng, YK: Yorkshire,DE: differentially expressed. (A) All stage-DE miRNA in TC and YK. The numbers marked in the overlapping areas show the stage-DE miRNAs in common. (B)The numbers of stage-DE miRNA in every comparable group in TC. The numbers above the blue are the numbers of up-regulated stage-DE miRNA. The numbers above the orange are the numbers of down-regulated miRNA. (C) The numbers of stage-DE miRNA in every comparable group in YK.
(TIF)

S5 Fig Interaction network of breed-DE miRNAs and breed-DE mRNAs. The network was constructed using 464 pairs of breed-DE miRNAs with breed-DE mRNAs. The green diamonds represent miRNA and the red circles represent mRNA. The nodes with bigger size had higher degree. TC: Tongcheng, YK: Yorkshire. Stages: 40, 55, 63, 70, and 90 days post coitum (dpc).
(PDF)

S1 Table Overview of sequencing data. (XLSX)

S2 Table Expression profiles of miRNAs divided into three categories. (XLSX)

S3 Table Target genes of miRNAs. (XLSX)

S4 Table Highly expressed miRNAs for cluster analysis. (XLSX)

S5 Table Functional annotation of the miRNAs belonging to cluster 0 in STEM. Ranked in an order of descending TPM value (average).
(XLSX)

S6 Table Differentially expressed (DE) miRNAs among different developmental stages. (XLSX)

S7 Table Functional annotation of stage-DE miRNAs. (XLSX)

S8 Table Breed-DE miRNAs between TC and YK. (XLSX)

S9 Table Data for building interaction network of breed-DE miRNAs and breed-DE mRNAs between TC and YK. (XLSX)

S10 Table The topological property of the networks with the breed-DE miRNAs and breed-DE mRNAs between TC and YK. (XLSX)

S11 Table Primers for plasmid construction and point mutation. (XLSX)
