# Supplementary material for: Comparative analyses of longissimus muscle miRNAomes reveal microRNAs associated with differential regulation of muscle fiber development between Tongcheng and Yorkshire pigs
Source: PLoS One. 2018 Jul 11;13(7):e0200445. doi: 10.1371/journal.pone.0200445 (PMC6040776; doi:10.1371/journal.pone.0200445)
Supplement: S1 Fig — (A) Breed-specific DE miRNAs found at any of the five developmental stages were respectively paired with their software-predicted gene/mRNA targets, based on the negative correlation (Spearman’s correlation coefficient<0.5) between the expression trends of the miRNA and the gene/mRNA during muscle development in TC and/or YK. (B) Breed-DE mRNAs (also between TC and YK) found at any of the five developmental stages were gathered. (C) The intersection of the set “breed-DE miRNA & target pairs” and the set “breed-DE mRNAs” were considered as the breed-DE miRNA & breed-DE mRNA pairs. (PPTX) [file pone.0200445.s001.pptx]

## Slide 1
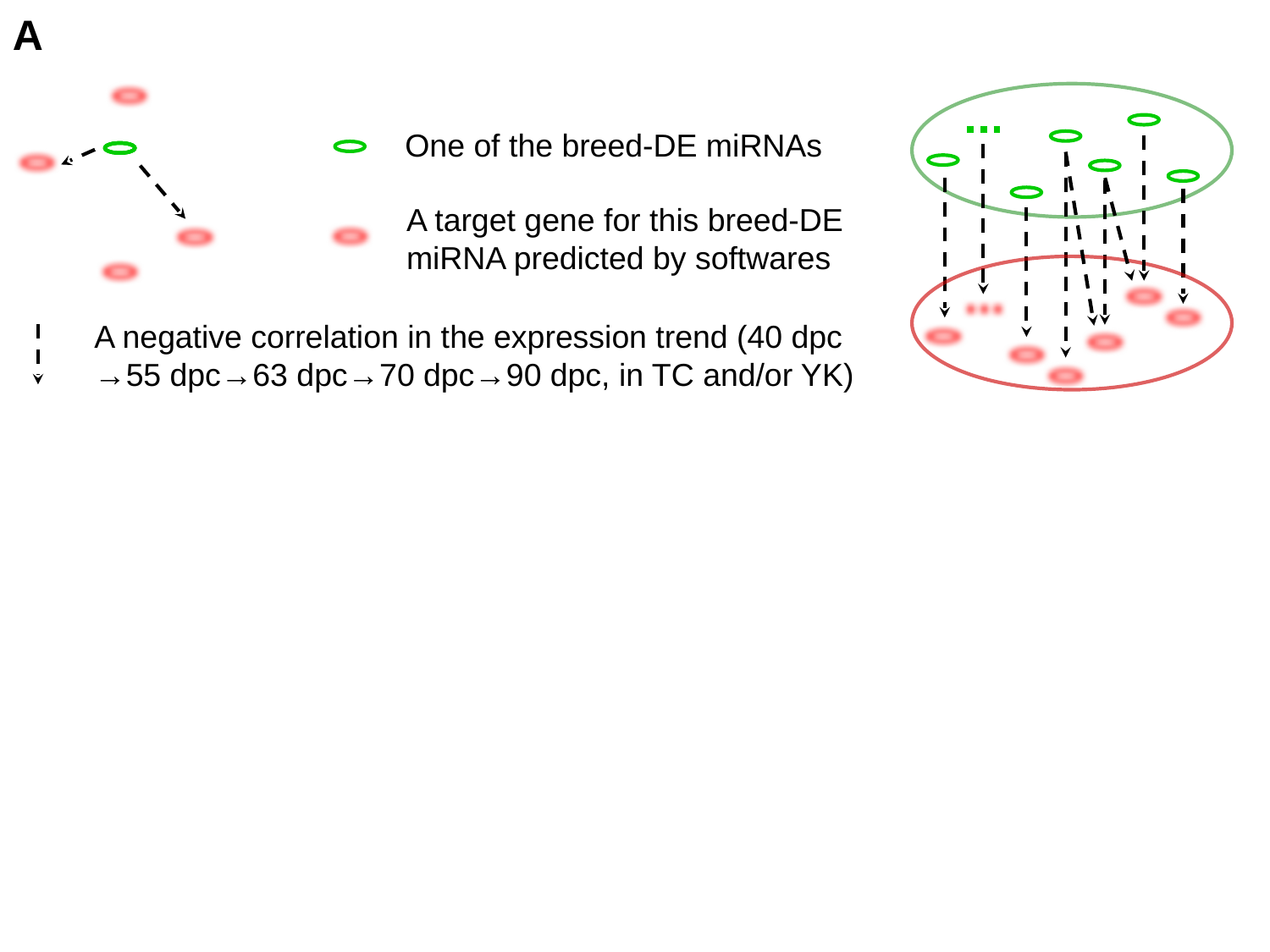

A
One of the breed-DE miRNAs
A target gene for this breed-DE
miRNA predicted by softwares
A negative correlation in the expression trend (40 dpc
→55 dpc→63 dpc→70 dpc→90 dpc, in TC and/or YK)

## Slide 2
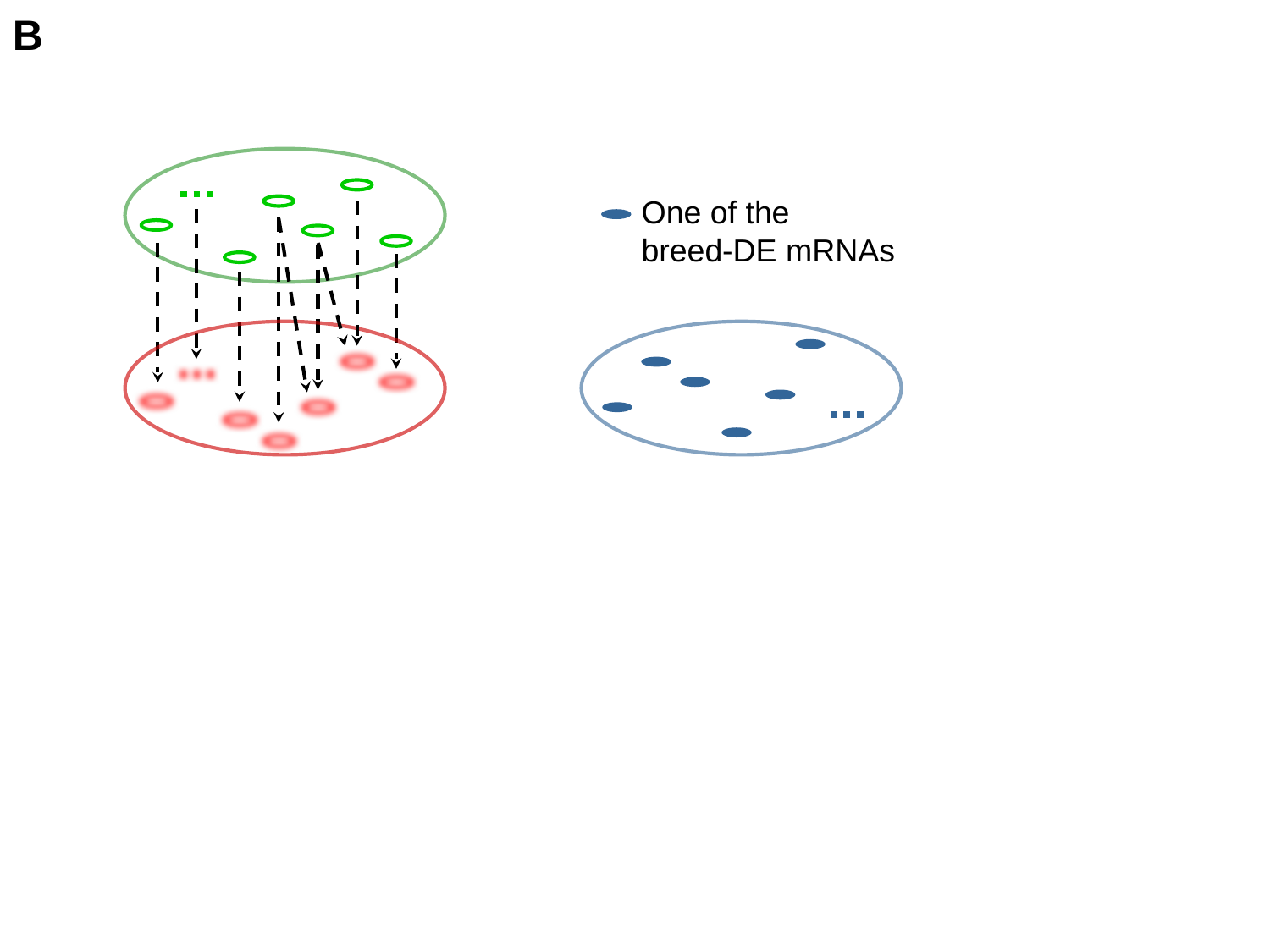

B
One of the
breed-DE mRNAs

## Slide 3
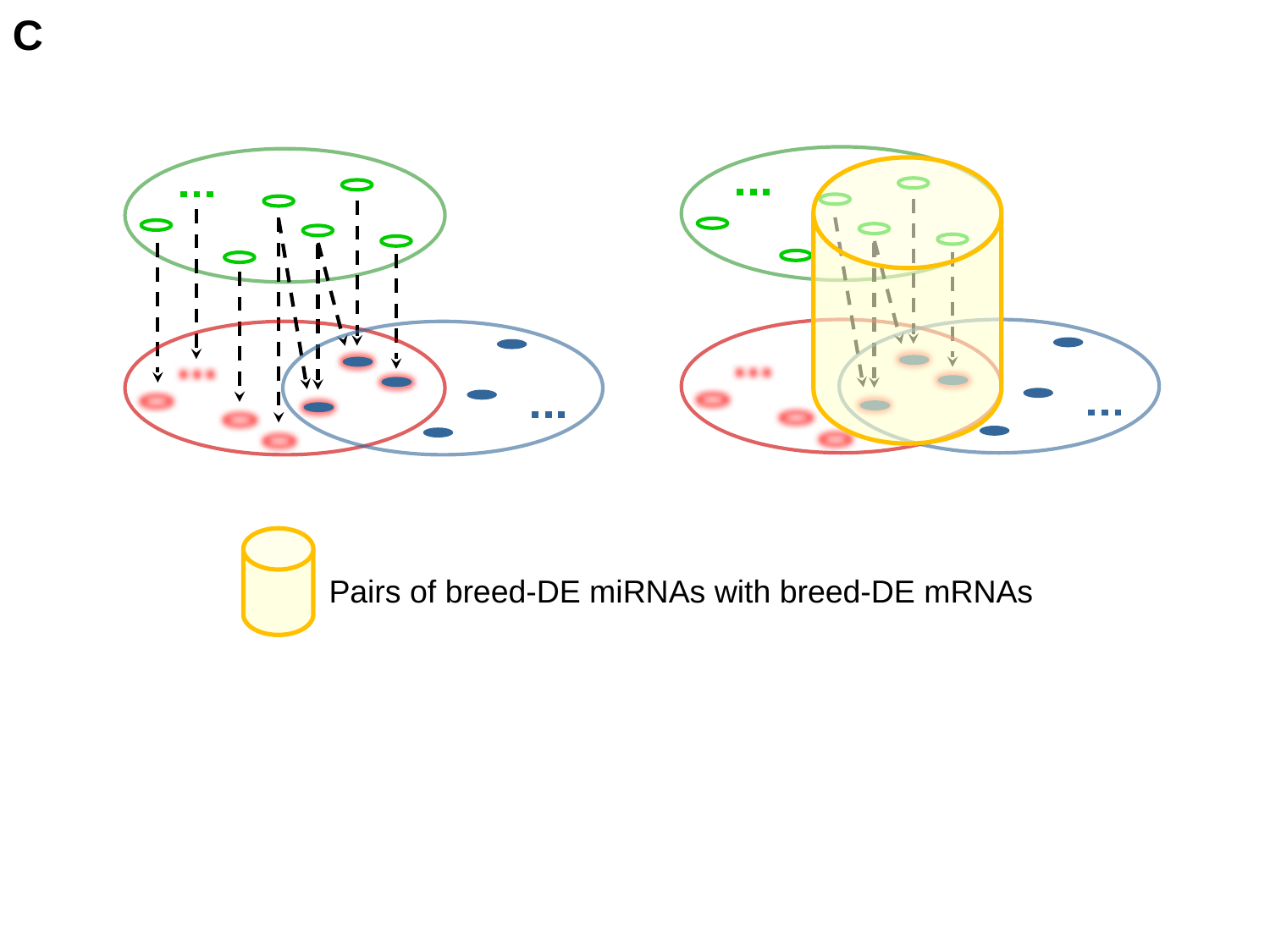

C
Pairs of breed-DE miRNAs with breed-DE mRNAs
