# Supplementary figures and images for: Comparative analyses of longissimus muscle miRNAomes reveal microRNAs associated with differential regulation of muscle fiber development between Tongcheng and Yorkshire pigs
Source: PLoS One. 2018 Jul 11;13(7):e0200445. doi: 10.1371/journal.pone.0200445 (PMC6040776; doi:10.1371/journal.pone.0200445)

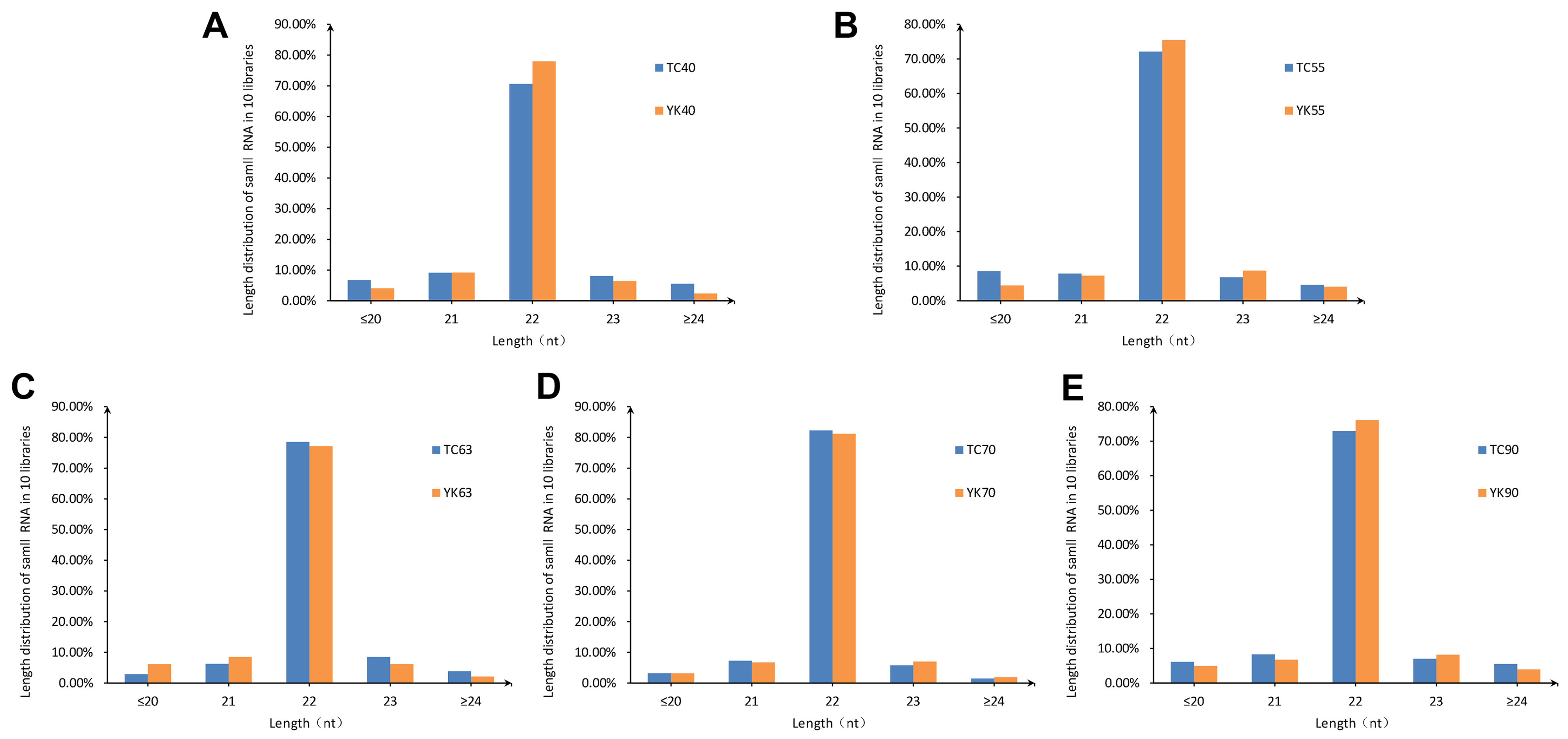

Supplement: S2 Fig — TC: Tongcheng, YK: Yorkshire. Stages: 40, 55, 63, 70, and 90 days post coitum (dpc) (TIF) [file pone.0200445.s002.tif]

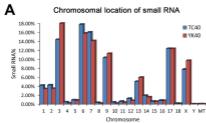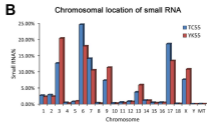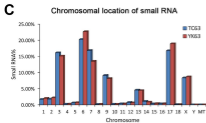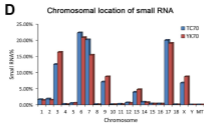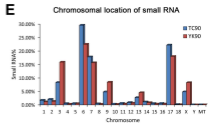

Supplement: S3 Fig — Chromosomal locations of small RNAs are displayed across 20 chromosomes and mitochondrial DNA (MT). TC: Tongcheng, YK: Yorkshire. (PDF) [file pone.0200445.s003.pdf]

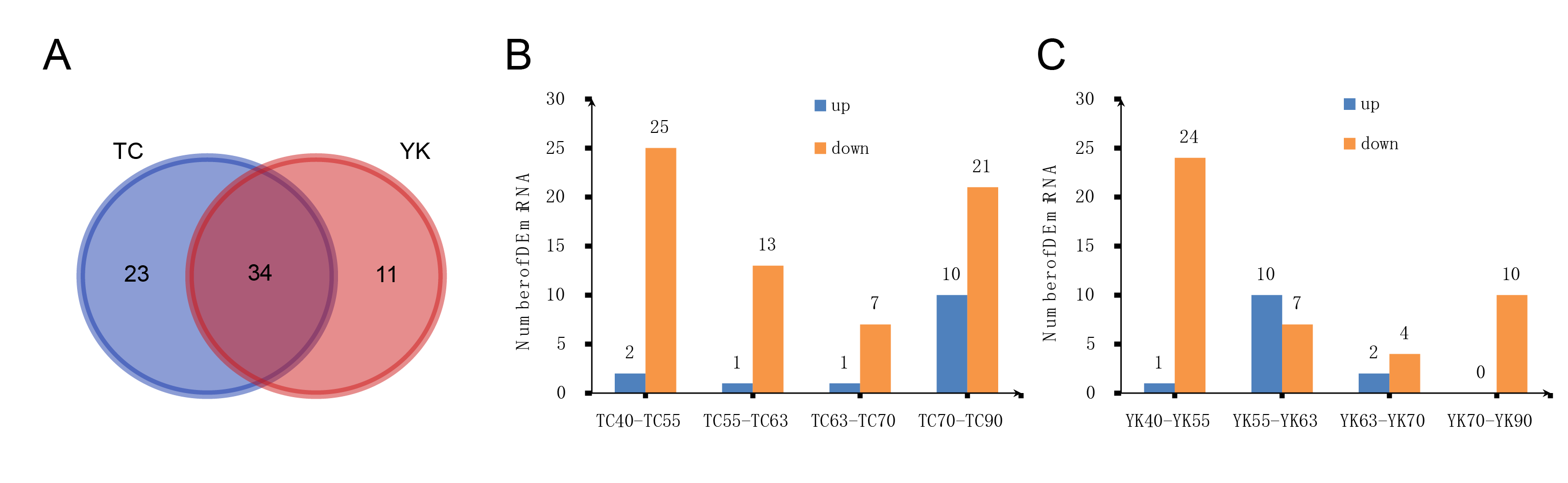

Supplement: S4 Fig — Number of differentially expressed miRNAs. TC: Tongcheng, YK: Yorkshire,DE: differentially expressed. (A) All stage-DE miRNA in TC and YK. The numbers marked in the overlapping areas show the stage-DE miRNAs in common. (B)The numbers of stage-DE miRNA in every comparable group in TC. The numbers above the blue are the numbers of up-regulated stage-DE miRNA. The numbers above the orange are the numbers of down-regulated miRNA. (C) The numbers of stage-DE miRNA in every comparable group in YK. (TIF) [file pone.0200445.s004.tif]

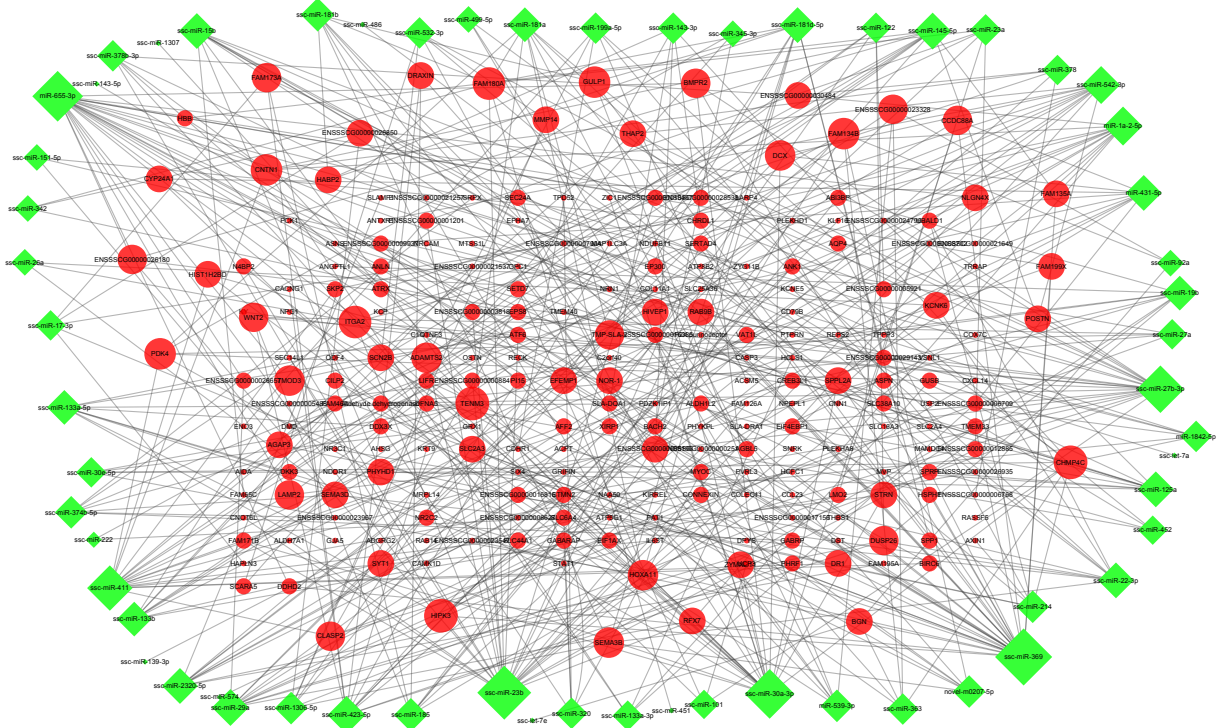

Supplement: S5 Fig — The network was constructed using 464 pairs of breed-DE miRNAs with breed-DE mRNAs. The green diamonds represent miRNA and the red circles represent mRNA. The nodes with bigger size had higher degree. TC: Tongcheng, YK: Yorkshire. Stages: 40, 55, 63, 70, and 90 days post coitum (dpc). (PDF) [file pone.0200445.s005.pdf]
